# Microbial amyloids in neurodegenerative amyloid diseases

**DOI:** 10.1111/febs.17023

**Published:** 2023-12-11

**Authors:** Timothy Sampson

**Affiliations:** ^1^ Department of Cell Biology Emory University School of Medicine Atlanta GA USA; ^2^ Aligning Science Across Parkinson's (ASAP) Collaborative Research Network Chevy Chase MD USA

**Keywords:** amyloid, microbiome, neurodegeneration, prion

## Abstract

Human‐disease associated amyloidogenic proteins are not unique in their ability to form amyloid fibrillar structures. Numerous microbes produce amyloidogenic proteins that have distinct functions for their physiology in their amyloid form, rather than solely detrimental. Emerging data indicate associations between various microbial organisms, including those which produce functional amyloids, with neurodegenerative diseases. Here, we review some of the evidence suggesting that microbial amyloids impact amyloid disease in host organisms. Experimental data are building a foundation for continued lines of enquiry and suggest that that direct or indirect interactions between microbial and host amyloids may be a contributor to amyloid pathologies. Inhibiting microbial amyloids or their interactions with the host may therefore represent a tangible target to limit various amyloid pathologies.

## Graphical abstract

Numerous microbes, including those indigenous to the human microbiome, produce amyloidogenic proteins. These proteins have critical functions in bacterial physiology and their lifestyles. Emerging evidence indicates that microbial amyloids also interact directly or indirectly with host‐derived amyloid proteins, such as those which underlie Parkinson's and Alzheimer's diseases. In this review, we discuss these interactions between microbial and host amyloid proteins and their contributions to neurodegenerative, amyloid diseases.

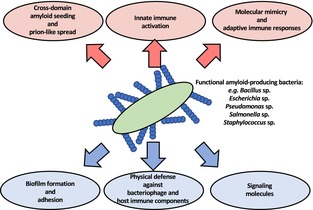

**Abbreviations**
⍺Syn: alpha‐synuclein
AD: Alzheimer's disease
APP: amyloid precursor protein
Aβ: amyloid‐beta
ALS: amyotrophic lateral sclerosis
Prp^C^
: cellular prion protein
CNS: central nervous system
GI: gastrointestinal
IM‐MS: Ion‐mobility spectrometry–mass spectrometry
IAPP: islet amyloid polypeptide
PD: Parkinson's disease
PSM: phenol soluble modulins
ROS: reactive oxygen species
SEVI: semen enhancer of viral infection
SAA: serum amyloid A
SOD1: superoxide dismutase 1
TLR2: toll‐like Receptor 2
TLR4: toll‐like Receptor
UPEC: urinary pathogenic *Escherichia coli*

## Introduction

A hallmark feature of many neurodegenerative diseases is the presence of aberrant protein aggregates in the brain and other organs. The accumulation of these protein inclusions is associated with neuronal dysfunction and disease progression. Such pathological proteinopathy is shared among neurodegenerative disorders as varied as Alzheimer's, Parkinson's, and infectious prion diseases [1]. As the primary constituent of these protein aggregates, amyloid proteins have emerged as crucial players in the etiopathogenesis of neurodegeneration. Experimental models have focused on the sufficiency of amyloid proteins to induce pathology and implicate their aggregation and prion‐like properties in disease etiology [2]. Emerging data within translational paradigms are providing support for anti‐amyloid interventions with various levels of efficacy in human trials [3].

Although traditionally associated with human diseases, amyloid proteins are evolutionarily conserved and prevalent in many organisms, including bacteria and other microbes [4]. Rather than pathological misfolding events, microbially derived amyloidogenic proteins have central roles in their biology and are often termed functional amyloids [5]. Commonly, these proteins have functions in surface attachment and creation of complex multicellular structures. There are now emerging data that demonstrate interactions between microbially derived amyloids and mammalian hosts that may be involved in the development and progression of neurodegenerative diseases. In this review, we delve into the intricate relationship between bacterial amyloids derived from indigenous and infectious organisms and their impacts on host physiology—focusing on outcomes relevant for neurodegeneration.

## Amyloidogenic proteins

**Fig. 1:**
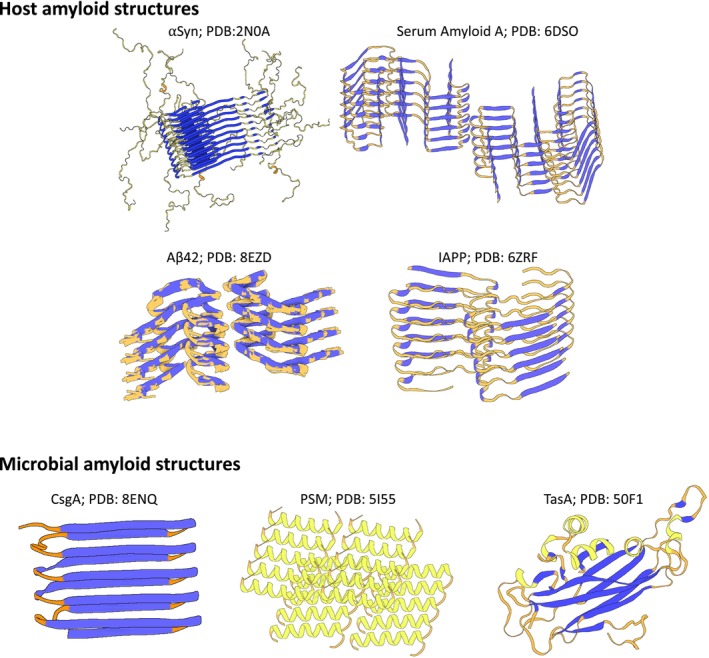
Representative structures of cross‐kingdom amyloid fibrils. Host and microbial‐derived amyloidogenic proteins share some features that give rise to fibrillar structures. ⍺‐synuclein (PDB: 2N0A), serum amyloid A (PDB: 6DSO), amyloid beta 42 (PDB: 8EZD), and islet amyloid peptide (PDB: 6ZRF) from mammalian hosts have been observed. CsgA (PDB: 8ENQ), PSM (PDB: 5I55), and TasA (PDB: 50F1) from microbes have been observed, with Staphylococcus‐derived phenol soluble modulins (PSMs) having a cross‐alpha, rather than cross‐beta, conformation. All structures acquired from public PDB repository, and color coded blue (beta sheet), yellow (alpha helices), and orange (coils), and constructed with protein imager (3dproteinimaging.com/protein‐imager).

Amyloidogenic proteins have the capacity to form structurally ordered protein aggregates most often characterized by a β‐sheet‐rich conformation [6, 7]. This conformation has a distinctive capacity to self‐assemble into highly stable, multimeric fibrillar structures, termed amyloids, which are very resistant to degradation [8, 9]. While these proteins are present in numerous compartments and cell types, they are perhaps most notable for their formation and deposition in the central nervous system (CNS). In the context of neurodegenerative diseases, the accumulation of amyloid deposits of specific compositions of these amyloid proteins (in conjunction with other protein species) in defined brain regions is a pathological hallmark of these conditions—for instance, amyloid‐beta (Aβ) in Alzheimer's disease, alpha‐synuclein (⍺Syn) in Parkinson's disease, and prion proteins in prion disorders, among numerous others (Table 1). Structures for many of these mammalian amyloidogenic proteins have been solved, both in monomeric and fibrillar forms [10, 11, 12, 13], and a nonexhaustive representation displayed in Fig. 1 [14].

**Table 1:** Neurological disease‐associated amyloidogenic proteins.

Disease	Primary amyloid protein	Clinical features
Alzheimer's disease	Amyloid beta Tau	Behavioral changes, characteristic cognitive decline and memory loss
Parkinson's disease	⍺‐synuclein	Hallmark motor disturbances (tremor, rigidity, bradykinesia) and autonomic dysfunctions
Familial amyloid polyneuropathy	Transthyretin	Peripheral neuropathy and sensory dysfunctions
Cerebral amyloid angiopathy	Amyloid beta Tau	Cerebral hemorrhaging
Prion disease	Prion protein	Progressive broad neurological dysfunctions
Huntington's disease	Huntingtin	Motor dysfunctions, cognitive decline
Amyotrophic lateral sclerosis	Superoxide dismutase 1 TAR DNA‐binding protein 43	Progressive paralysis
Frontotemporal dementia	Tau TAR DNA‐binding protein 43	Behavioral changes, cognitive decline

In humans and other mammals, these proteins natively exist in a nonamyloid form with critical roles in physiology. Alpha‐synuclein (⍺Syn), as one example, is involved in synaptic vesicle formation and function [15, 16]. Amyloid Precursor Protein (APP), which is cleaved into Aβ, is also believed to have roles in synapse development [17, 18]. Emerging data even implicate both APP and ⍺Syn in immune regulation, with APP showing antimicrobial activity and ⍺Syn involved in the resistance to neurotropic viral infections [19, 20, 21, 22]. The current body of evidence in humans and experimental models indicates that the pathogenic properties of amyloidogenic proteins likely manifest due to direct, detrimental properties of the insoluble amyloid forms. However, a nonmutually exclusive hypothesis has been proposed that the loss of native function of the amyloidogenic protein, due to the misfolded conformation or accumulation into aggregates, may also underlie some disease etiologies [23, 24]. This is perhaps exemplified in amyotrophic lateral sclerosis (ALS), where loss of superoxide dismutase 1 (SOD1) native activity (due to aggregate formation) leads to pathogenesis [25]. However, knockout models of synuclein (including triple alpha‐, beta‐, and gamma‐, synuclein) and APP do not appear to recapitulate the hallmark neurodegeneration associated with Parkinson's or Alzheimer's diseases, respectively [26, 27].

## Pathogenic mechanisms of amyloid proteins in disease

A broad swath of mechanisms by which amyloidogenic proteins trigger disease and promote neurodegeneration has been proposed. No single property is likely solely causative for disease. In combination, however, these properties can result in etiopathogenesis. It is also important to note that the precise structural form of each amyloid that imparts these various effects is not yet abundantly clear. Amyloid pathology appears not only as large fibrillar aggregates but also as smaller oligomeric forms [28]. These smaller molecular weight species may be generated by breakdown of the larger fibrils or formed during the process of fibril formation. More nuanced structural differences between identical amyloid sequences may also give rise to very different pathogenic properties [29]. Dependent on the protein species, it does appear that each of these forms, or strains, can impart differential effects on pathological processes [30, 31, 32].

One key pathogenic effect of amyloid aggregates is on mitochondrial function. Aggregates can directly interact with mitochondria, leading to energy deficits and increased production of reactive oxygen species [33, 34, 35, 36, 37, 38, 39]. In energy‐intensive cells, such as neurons, these effects can be exacerbated resulting in oxidative stress and neuronal damage [33, 34]. Some neuron‐types appear most sensitive to these damages, such as highly arborized dopamine neurons [40, 41]. As one example, ⍺Syn aggregates impair mitochondrial trafficking and turnover which is sufficient to result in loss of sensitive dopaminergic neurons affected during Parkinson's disease (PD) [35]. Similar effects are observed with Aβ aggregates which can accumulate in mitochondria and inhibit ATP production [34] Amyloids can also greatly impair proteostasis, resulting in dysregulated protein turnover by autophagosomal and proteosomal clearance pathways [34, 38, 42]. Aβ and ⍺Syn both inhibit the ubiquitin‐proteosome system and can starkly impair autophagy [32, 34, 42, 43, 44, 45]. Disruption of cellular proteostasis increases the propensity for other proteins to misfold and ultimately impact cellular toxicity and pathological spread.

The impacts on mitochondrial activity and proteostasis can indirectly lead to immune activation through release of damage‐associated signals, but amyloids can also directly promote inflammatory signaling in both brain‐resident and peripheral immune cells [46, 47]. Both Toll‐like Receptor 2 (TLR2) and TLR4 are present on brain‐resident glia and other immune (and non‐immune) cells, including neurons. Interestingly, both TLR2 and TLR4 appear to be responsive to a number pf amyloid proteins directly [47, 48, 49, 50, 51, 52]. A diverse array of amyloid proteins, including ⍺Syn, Aβ, islet amyloid peptide, prion protein, and transthyretin all trigger TLR activation and inflammatory cytokine production [47, 51, 53, 54, 55, 56, 57, 58, 59]. Increasing the inflammatory tone of the cellular environment may create a cycle of dysfunction, through production of reactive oxygen species and other cellular or neuronal impairments. These signals may both potentiate cell death signals but also promote an environment that increases the propensity of amyloid misfolding events to occur [60].

A final characteristic feature of amyloid proteins is their ability to propagate and spread across anatomical sites [1, 2, 7]. Exemplified by prion proteins, misfolded amyloid forms are capable of templating native structures into amyloids [61]. This process seeds aggregate formation across connecting cells and anatomical circuits resulting in a spread of pathology from an initial site. For nonfamilial prion diseases, propagation of amyloid from the GI tract due to ingested prion‐infected tissues, results in the classical neuropathologies of prion disorders such as scrapie, kuru, and Creutzfeld Jakob disease [61]. In other amyloid disorders, this propagation is thought to underlie disease progression, as new circuits become impacted by pathology. Braak staging of PD and Alzheimer's disease (AD) serves as a model to overlay a prion‐like propagation with disease progression in humans [2, 62, 63]. Experimental systems using purified proteins, *in vitro* cell culture systems, rodent models, and nonhuman primate models, largely support the ability of amyloidogenic proteins, such as ⍺Syn, Aβ, and tau to spread within the brain and from peripheral sites, such as the GI tract [2, 64, 65, 66, 67, 68, 69]. Given the fact that prion protein canonically spreads from the GI tract to brain following oral exposure in non‐familial forms of disease [70, 71], it is an enticing hypothesis that these other amyloid diseases may do so as well. However, despite the experimental evidence in models, it remains unclear if peripheral prion‐like propagation of amyloids such as ⍺Syn and Aβ underlies their respective disease etiologies or progression in humans.

## Bacterial amyloids and their functions

Intriguingly, amyloid proteins are not exclusive to the realm of neurodegenerative diseases, detrimental pathologies, or even mammals and other animals. Proteins with these discrete structural folding capacities are genetically encoded across domains of life and are highly abundant in various microbial, particularly bacterial species [72, 73]. Bacterial amyloids do not appear to arise due to misfolding events and instead their transformation into the amyloid conformation is a highly regulated and evolutionarily conserved process. Bacterial amyloids ultimately play important roles in microbial physiology, which are reliant upon their ability to form self‐assembling, fibrillar structures [4]. These proteins are known to contribute to biofilm formation, mediate cell adhesion, and provide structural stability to bacterial communities, among other functions. Given these roles, the term ‘functional amyloid’ has arisen to differentiate between these physiological roles and the pathogenic properties of misfolded mammalian amyloids.

Functional microbial amyloids are a highly conserved structure across diverse species (Table 2), and representative comparisons of solved structures displayed in Fig. 1 [74, 75, 76]. Staining‐based methods for identifying amyloids suggests that up to 40% of detectable bacteria generate secreted/extracellular amyloid proteins [77, 78]. While the exact sequences (both protein and nucleic acid) differ among diverse microbial amyloids, sequencing approaches validate the staining and culture‐based observations [72]. Homologs of the prototypical microbial amyloid, curli, are present in the vast majority of Enterobacteriaceae and orthologous amyloids are present in evolutionarily distinct taxa including Bacteroidetes and Firmicutes [72]. Computational and experimental evidence shed light on the sequence requirements for amyloidogenesis and are continuing to identify microbial proteins with intrinsic amyloidogenic properties [73]. Given the sequence diversity, microbial amyloids likely evolved multiple times, and prototypical microbial amyloids include curli/CsgA in *Escherichia coli* and other Enterobacteriaceae, FapC in *Pseudomonas* sp., and TasA in *Bacillus subtilis*. While bacterial species represent an important amyloid encoding reservoir, various fungi and virus can also produce proteins with amyloidogenic properties that are important for their functions [79].

**Table 2:** Representative functional microbial amyloids.

Amyloid	Microbe	Function
Curli (CsgA)	Enterobacteriaceae and others	Biofilm formation, adhesion, cell surface stability
Phenol soluble modulins	*Staphylococcus* sp.	Biofilm formation and dispersal
FapC	*Pseudomonas* sp.	Biofilm formation
TasA	*Bacillus* sp.	Biofilm formation, cell surface stability
Type IV pili CarD	*Mycobacterium* sp.	Adhesion Transcription factor
Sup35	*Sacchromyces cervisease*	Translation termination
Microcin E492	*Klebsiella* sp.	Pore‐forming toxin
Rho	*Clostridium* sp.	Transcription factor
Chaplins	*Streptomyces* sp.	Hydrophobic surface coating

The importance of functional amyloids to the lifestyle of those organisms encoding them is perhaps best exemplified by the curli amyloid. This component of the extracellular matrix is extremely prevalent in Enterobacteriaceae and across diverse strains of *E. coli* [72]. So much so, the operon encoding curli and its regulatory and biogenesis machinery are a component of the core *E. coli* genome [80, 81]. These core operons are nearly invariable between strains of *E. coli*‐irrespective of their diverse lifestyles [80]. Further evidence of their critical importance to microbial physiology is the strict regulation of amyloid production. As observed in eukaryotic systems, oligomeric intermediates in the pathway toward fibril formation are often highly toxic [28, 82]. This continues to be true in prokaryotic systems as well. Microbial amyloids can interact with and disrupt membranes leading to cell death, if formed at inappropriate times or locations [83]. Therefore, amyloid production has evolved an exquisitely controlled and segregated regulatory process, which prevents oligomers from forming and allows for rapid fibril formation at the appropriate location and time [83]. For instance, CsgA, the major subunit of curli fibers, are bound in a nonamyloid form to an array of specific chaperones as it is produced and transported across the inner membrane, through the periplasmic space, and released through the outer membrane to allow seeding into fibrils extracellularly (stochastically or at specific nucleation sites such as CsgB or fibrilized CsgA) [83, 84, 85].

This is further regulated by careful response to particular environmental signals to drive expression of curli structural proteins and synthesis machinery with functional amyloid being produced during key physiological conditions. Various factors, such as environmental cues, growth conditions, and protein chaperones, can influence the kinetics and regulation of bacterial amyloid formation. For instance, certain environmental stresses, such as temperature or exposure to antimicrobial agents, can trigger amyloidogenesis as part of the bacterial stress response [83].

Across diverse species, functional amyloids have a number of direct and distinct roles in microbial physiology. Microbial amyloids are often secreted into the environment where they associate with the microbial or environmental surfaces, a property which is central to many of their functions [4, 83, 86, 87]. Upon secretion, some amyloids provide microbes the ability to closely associate with both biotic and abiotic surfaces where they are found in complex extracellular matrices [88]. These scaffolds are highly adherent, a property that in some cases is based upon the structure of the amyloid itself, rather than solely sequence, given the diversity of orthologs encoded by microbes [72, 89]. This allows microbes to adhere to host tissues and initiate colonization or infection, or to colonize dynamic and diffusion prone environments [4, 5].

Once adhered, amyloid proteins allow microbes to remain sessile and form complex, organized, and multicellular biofilm structures. Biofilm communities, often populations of diverse bacterial species and not mono‐cultures, are held together physically by the extracellular matrix [90, 91]. Bacterial amyloids form an abundant proteinaceous component creating a scaffold for other constituents, such as DNA and various carbohydrates to adhere. Within the intricate biofilm structure, microenvironments create gradients of local molecular signals and ultimately trigger highly diverse metabolic programs within the organisms present in distinct regions [90, 91]. This allows the community to survive numerous environmental insults, including host immune pressures during infection [87]. Indeed, biofilm and biofilm‐like structures are found associated with numerous infectious conditions—of which microbially produced amyloids are central to their composition [92].

As components of the extracellular matrix, bacterial amyloids also provide physical defense against bacteriophage predation. Amyloid coatings have been shown to prevent phage from successfully adsorbing and inserting into the bacterial host cell envelope—limiting there infectious cycle [93]. Similar structural properties of amyloids also affect the ability of other molecules to directly bind the bacterial envelope. This includes preventing the interaction with immune components during colonization or infection with animal hosts. Some bacterial amyloids have capacity to prevent host peptide adsorption and complement binding, successfully providing a barrier against immune defenses [94, 95, 96].

While these structural attributes are well‐studied, other functions of microbial amyloids are observed. Cleavage products of microbial derived amyloids have also been observed to act as signaling molecules and even toxins against other species competing in specific environments [88, 95, 97]. One described example of amyloid proteins serving as signals has been observed with the *Bacillus* sp. amyloidogenic protein, TasA. While this protein has an important role in the extracellular matrix, it also acts as a signaling molecule, detected by surface receptors within other cells in the community to regulate further adhesion and motility phenotypes as a whole [98]. Intracellularly, some bacterial proteins involved in central processes such as replication and transcription take on an amyloid conformation as part of their activity [99, 100]. It is possible that cellular environments that modulate the amyloidogenic conformation may thus regulate their activity. For those microbial amyloids with unknown functions, given their immense evolutionary conservation, they very likely have important roles in microbial physiology. Interestingly, some microbial amyloids are able to complement those of other species in their function—allowing mixed amyloid biofilm matrices to form from direct cross‐seeding interactions [101]. This suggests that these diverse proteins can also have important interactions with structurally similar orthologs.

## Cross‐domain amyloid interactions

Over the past decade, emerging evidence has demonstrated interactions between distinct amyloidogenic proteins and suggests the capacity for certain amyloid protein species to influence the accumulation and/or amyloidogenesis of diverse, nonidentical amyloid proteins. Heterologous interactions have been reported between the mammalian proteins ⍺Syn, Aβ, Tau, and PrP^C^ [45, 64, 102, 103, 104, 105, 106, 107, 108, 109, 110, 111]. This includes observations through a range of experimental systems. Various animal models have demonstrated that administration of amyloids of one protein can accelerate the pathological aggregation of other amyloids in the system. For instance, ⍺Syn injected into PrP^C^ mice results in significantly accelerated PrP^C^ pathology [102]. In human incidences of amyloid diseases, copathologies are often apparent. Amyloid aggregates in postmortem human brains show nonhomogenous compositions of amyloid proteins in numerous neurodegenerative diseases [108, 110]. While the mechanism of these observations are likely varied, cross‐seeding as evidenced by *in vitro* biochemical assessments is one possibility [104]. There is biochemical evidence that direct seeding interactions can occur, whereby one amyloid protein is able to directly template the misfolding of another, or accelerate the fibrillar extension, in a prion‐like fashion [103, 106, 112]. Indirect mechanisms may also occur to induce co‐pathologies and coaggregates, such as inhibiting proteostasis and promoting inflammatory environments. However, cross‐interactions between diverse amyloids are not universal [104]. Understanding the structural or sequence specificity that allows some amyloid proteins to trigger the accumulation or cross‐seed nonorthologs is of high interest in the field. Nonetheless, as a whole, these data suggest that certain mammalian amyloids can interact with other non‐identical proteins and accelerate pathology.

In line with this thinking, it is therefore possible that the structurally similar microbial amyloids may therefore also interact with diverse mammalian amyloids and perhaps modulate their conversion. Indeed, mounting evidence suggests that bacterial amyloids could interact with amyloid proteins associated with neurodegeneration and modulate their aggregation kinetics and toxic properties (Table 3). The first observations of this phenomenon were seen following the administration of either curli amyloid‐producing *E. coli* (heat‐inactivated) or purified curli fibrils into wildtype mice under a chronic inflammation paradigm [113]. Exposure to these microbial amyloids resulted in the increased prevalence of splenic serum amyloid A (SAA) pathology, demonstrating for the first time that microbial amyloids could trigger mammalian amyloid pathology [113]. Interestingly, the same study also exposed mice to amyloids derived from *Saccharomyces cerevisiae* (protein Sup35) as well as silk fibrils from silk moths, and also observed increased prevalence of SAA deposition [113]. These data provided the foundation to begin exploring the broader capacity of environmental and microbial amyloids to contribute to and/or accelerate mammalian amyloid pathology.

**Table 3:** Interactions between microbial and disease‐associated amyloids.

Microbial amyloid	Disease amyloid	Interaction	Model systems studied
CsgA	*aSyn*	Acceleration of amyloidogenesis	Mice, rats, *C. elegans*, *in vitro* protein
	*Abeta*	Acceleration of amyloidogenesis	*In vitro* protein
	*IAPP*	Acceleration of amyloidogenesis	*In vitro* protein
	*SEVI*	Acceleration of amyloidogenesis	*In vitro* protein
	*HTT*	Acceleration of amyloidogenesis	*C. elegans*
	*SOD1*	Acceleration of amyloidogenesis	Mice, *C. elegans*
	*Tau*	No effect	*In vitro* protein
FapC	*aSyn*	Acceleration or Inhibition of amyloidogenesis	*In vitro* protein
PSM	*aSyn*	Acceleration of amyloidogenesis	*In vitro* protein

### Microbial amyloid interactions *in vitro*

Building on this observation, the breadth of these cross‐domain interactions and those mechanisms by which microbial amyloids may interact to promote amyloidogenesis are beginning to be explored. Similar to mammalian amyloids, microbial amyloids have shown the ability during *in vitro* biochemical assays to directly cross‐seed mammalian amyloids [74, 114, 115, 116]. For instance, purified CsgA accelerates the amyloidogenesis of Aβ *in vitro* [74, 114]. Other amyloid proteins, including ⍺Syn, islet amyloid polypeptide (IAPP), and semen enhancer of viral infection (SEVI) also show enhanced amyloidogenesis in the presence of CsgA *in vitro* [114, 115, 117]. Interestingly, a pathogenic form of Tau did not show enhanced amyloidogenesis in the presence of CsgA, suggesting specificity to these interactions [115]. Not all interactions result in acceleration or enhanced amyloidogenesis *in vitro*. For instance, transthyretin results in inhibition of CsgA‐mediated amyloidogenesis [118].

The potential mechanisms by which CsgA may interact to accelerate amyloidogenesis *in vitro* and lead to enhanced aggregation are likely varied. However, given the nature of these described assays using purified forms of the various proteins, direct interactions are possible. This could include cross‐seeding or enhancement of elongation, for instance, but the precise nature of their interactions leading to enhancement of amyloidogenesis is still somewhat unclear. Surface plasmon resonance assays were unable to reveal a stable and direct interaction between ⍺Syn and CsgA monomers, possibly explained by technical limitations at the time [115]. More recently, using Ion‐mobility spectrometry–mass spectrometry (IM‐MS), a weakly bound 1 : 1 CsgA:⍺Syn complex was observed suggesting that indeed direct binding between these proteins seeds ⍺Syn amyloidogenesis [117]. Interestingly, quicker amyloid forming orthologs or mutants of CsgA are less able to catalyze ⍺Syn aggregation, compared to slower fibril forming CsgA species [117]. These slower forming CsgA species appear to more readily move between dimeric and monomeric forms, rather than continued large fibril formation, suggesting that they are more likely to be present in a monomeric form that can interact with ⍺Syn, promoting seed formation [117]. Transient interactions between chaperone proteins of CsgA are necessary to prevent it from forming toxic amyloid oligomers premature to its release into the extracellular space [84, 85]. Similar low affinity interactions between these chaperones and mammalian amyloids can differentially result in acceleration or inhibition of ⍺Syn (CsgE or CsgC, respectively) but not interact with Aβ (CsgC) *in vitro* [85]. This further adds to the plausibility of shared structural features that dictate the interactions between diverse amyloids across biological domains.

Other bacterial amyloids have demonstrated *in vitro* interactions with ⍺Syn as well. The Pseudomonad amyloid, FapC, involved in biofilm formation, is capable of enhancing ⍺Syn amyloid formation [116]. Similar to CsgA, this property is modulated by the intrinsic ability of FapC to form self‐amyloids. Conversely, mutations in FapC that prevent self‐assembly have also been shown to inhibit ⍺Syn aggregation [116], further extending the hypothesis of direct formation of cross seeding events between amyloid‐competent proteins. Phenol‐soluble modulins (PSM) are amyloidogenic proteins derived from *Staphylococcus* sp. that participate in biofilm formation and immune modulation also have been observed to accelerate ⍺Syn amyloidogenesis *in vitro* [119]. Interestingly, these proteins take on a cross‐alpha fibrillar structure (Fig. 1) [76], different from the cross‐beta amyloid conformation of CsgA and curli. While these data *in vitro* support the capacity of microbial amyloids to broadly accelerate mammalian amyloidogenesis, possibly through direct and seeding interactions, emerging data highlight the ramifications of these potential interactions in *in vivo* model systems.

### Microbial amyloid interactions *in vivo*

All animals, including humans, are colonized on environmentally exposed surfaces with a plethora of microbial organisms, the microbiome. The majority of these organisms reside in the GI tract. Over the past few decades, a number of experimental and clinical data implicate these organisms in numerous aspects of health and disease [120, 121]. This growing body of evidence indicates contributions of these organisms to neurological disease, in particular neurodegenerative diseases with hallmark amyloid pathologies [122]. As many of the organisms differentially associated with these outcomes produce a functional amyloid, it is interesting to speculate that the potential cross‐domain interactions between microbial and mammalian amyloids may have direct consequences on amyloid diseases.

Exemplified in more recent studies, CsgA and full‐length curli proteins have been shown to accelerate ⍺Syn pathology in a few model systems. First, oral exposure to curli‐producing *E. coli* in an aged rat model of synucleinopathy resulted in significantly enhanced ⍺Syn pathology [123]. Even through this more transient oral paradigm, simply being exposed to curli in the GI tract could accelerate amyloidogenesis in both intestinal and brain tissues [123]. Exposure to an isogenic curli‐deficient did not result in significant pathology, strongly suggesting that curli itself is responsible for triggering or accelerating ⍺Syn accumulation, rather than the potential stimulation by lipopolysaccharide also produced by *E. coli* [123]. Independently, mono‐colonizing curli‐producing or deficient *E. coli* into the GI tract of germ‐free Thy1‐hSNCA mouse model of synucleinopathy recapitulated this enhanced pathology in the gut and brain, as well as exacerbated hallmark behavioral outcomes in the model [115]. Thus, curli amyloid production in the GI tract was sufficient to drive accelerated ⍺Syn pathologies across model systems. Amyloid‐producing bacteria found outside of the GI tract have also been suggested to impact amyloid pathology. Recently, in a urinary tract infection model, urinary pathogenic *E. coli* (UPEC) was shown to induce synucleinopathy in infected tissues [124]. UPEC strains of *E. coli* produce curli amyloids that are thought to be important to their ability to colonize the urinary tract [125]. While not tested directly, it is possible that the curli amyloids produced by UPEC contribute to local synuclein accumulation into insoluble forms.

Additionally, an unbiased screen in *Caenorhabditis elegans* has further identified CsgA as necessary for *E. coli* to induce neurodegeneration. It was observed that nematodes fed curli‐producing, but not curli‐deficient *E. coli* also displayed significant ⍺Syn aggregation in *C. elegans* producing human synuclein [126], validating original observations [123]. Interestingly, not only was CsgA capable of accelerating synuclein accumulation in the nematodes, but also triggered SOD1 and huntingtin amyloid accumulation in *C. elegans* producing human forms of these amyloidogenic proteins [126]. SOD1 amyloid pathology and neurodegeneration was also recently found to be accelerated in curli‐exposed and colonized mice overproducing a pathogenic form of SOD1 [127]. Altogether these data strongly suggest that the presence of curli or other orthologous and non‐orthologous microbial amyloids may contribute to pathological aspects not only of ⍺Syn but other amyloid‐mediated diseases.

## Other influences by microbial amyloids on neurodegeneration

The various rodent and nematode experimental paradigms do demonstrate that microbial amyloids (namely CsgA/curli) are necessary and sufficient to induce enhanced amyloid (mostly ⍺Syn) pathology *in vivo*. Yet, how this pathology becomes triggered is still unclear. The biochemical, *in vitro* evidence suggests that this may be due to direct, cross‐seeding interactions between microbial and mammalian amyloids, accelerating their amyloidogenesis. However, at the time of this writing, direct interactions have not been observed *in vivo*. Microbial amyloids have the potential to act on other relevant systems that may therefore contribute to amyloid pathology in a non‐mutually exclusive way.

Like mammalian amyloids, microbial amyloids are significant triggers of immune signaling. Curli and other microbial amyloids signal through TLR2 and TLR4, leading to various inflammatory responses [87, 128]. It may be that increased amyloid deposition during colonization or infection by curli‐producing microbes *in vivo* is an effect of alterations in inflammatory tone. An increased inflammatory environment increases ROS and impacts proteostasis, and thus alter the propensity of amyloids to accumulate. There is also substantial evidence that certain microbial amyloids, namely curli, when derived from complex biofilm structures (for instance, with free DNA associated) trigger autoimmune responses [92, 129]. In particular, these microbial amyloid complexes induce the production of auto‐antibodies against DNA, a hallmark of systemic lupus erythematosus [129]. It suggests that perhaps infection (or colonization by) amyloid producing bacteria may contribute to the etiological risk of this disease.

Whether the production of auto‐immune responses contribute to neurodegenerative amyloid diseases is not yet clear. Auto antibodies against disease‐associated amyloids, such as ⍺Syn, Aβ, Tau, and SOD1 have been reported [130, 131, 132, 133, 134, 135]. However, it is unknown whether these are pathogenic and contribute to disease. An alternative is that their presence is an epiphenomenon and marker of an ongoing neurodegenerative process. In addition to auto‐antibodies, auto‐reactive T cells that recognize amyloid epitopes appear during synucleinopathies and Alzheimer's disease [136, 137, 138, 139]. In experimental systems, there is emerging evidence that these T cells may be pathogenic and drive aspects of disease pathology [46, 140, 141, 142], but observations in human amyloid diseases are still in their infancy. It may be that microbial amyloids serve as mimics and such auto autoimmune responses due to structural similarities in conjunction with environmental contexts [143, 144, 145]. However, at the time of this writing, there is no evidence for cross‐reactivity between disease‐associated and microbially derived amyloids by the autoantibodies and/or autoreactive T cells found during amyloid diseases. Nonetheless, given the capacity for direct cross‐seeding and the structural similarities between the proteins, the hypothesis of molecular mimicry between microbial and mammalian amyloids is plausible and interesting to consider.

## Relevance within the gastrointestinal tract

Interactions between human hosts and microbial amyloids largely occurs in the context of certain infectious organisms or members of the indigenous microbiomes colonizing various body sites largely outside the CNS. If microbial amyloids produced at these distal body sites, for instance the GI tract, are indeed capable of triggering pathologies in more distant locations, how the microbe, it' is amyloid, and/or a signal‐derived from the amyloid are trafficked is not yet fully understood.

Certainly, influencing immune responses (as described in the prior sections) may be one way in which microbial amyloids modulate the host environment toward pathology. This could occur in a more systemic fashion, promoting amyloid deposition at sites sensitive to an increased inflammatory tone. It could also be more direct; microbial amyloid‐specific immune responses are trained in the GI tract or at sites of infection and lead to immune responses that promote pathology. An immune‐mediated mechanism is not mutually exclusive to a cross‐seeding, but does provide a hypothesis to explain anatomically distant effects of microbial amyloids.

While the biochemical evidence does suggest an ability to cross‐seed between microbial and host amyloid, this hypothesis necessitates that the two proteins become co‐localized at some point during colonization or infection. At the time of this writing, co‐localized microbial and host amyloid *in vivo* has not been observed. Evidence is currently lacking that conclusively demonstrates that microbial amyloids or the amyloid‐producing microbes are trafficked to the CNS, for instance. Even more locally, having both the microbial and mammalian proteins in the same physical space at the same time remains a conceptual challenge. In the GI tract, enteric neurons and enteroendocrine cells within the epithelial layer produce ⍺Syn as an example, but it is largely enriched intracellularly at synapses [146]. Microbial amyloids derived from the GI tract would need to be internalized (or the mammalian amyloidogenic protein released extracellularly) into the intestinal lumen, and then reabsorbed. In the case of orally acquired prion disease (e.g., kuru, CJD), PrP^C^ appears to be preferentially taken up by M cells of the intestinal epithelium, even if those cells are not themselves producing PrP^C^ [147]. Other immune cells of the GI tract may also sample bacterial and environmental amyloids from the intestinal lumen. Indeed, particular immune cells have been observed to traffic amyloid‐competent proteins, such as Aβ, from peripheral sites to the CNS which could seed pathology [148]. ⍺Syn‐producing enteroendocrine cells have also been observed to take up luminal antigens [146, 149]. However, it is not known whether microbes or their products are directly sampled by enteroendocrine cells, or whether these phenomena may occur with microbial amyloids sampled from environmentally exposed surfaces, such as the gut.

Seeding pathology at one site is experimentally validated to result in pathological prion‐like spreading of ⍺Syn and other amyloid proteins [2, 65, 66, 150, 151, 152]. This could allow for microbial amyloids to seed or otherwise trigger a pathogenic amyloid event in the GI tract. For instance, ⍺Syn amyloids seeded in the GI tract have been observed to propagate to the CNS, to various extents in rodent models, through both the vagus nerve and spinal cord [65, 66, 150, 151, 152]. In human cohorts, vagotomy and appendectomy have been linked to decreased risk of synucleinopathy [153, 154], suggesting that this neuronal route and microbial reservoir may contribute to aspects of disease. Thus, potentially local interactions between microbial amyloids and the host in the GI environment could be sufficient to trigger disease. While some have proposed that certain indigenous microbes can be trafficked to the CNS, there is currently no evidence that microbial amyloids themselves are present in the brain.

## Ramifications on disease—etiology and therapeutics

The ability of functional, microbial amyloids to facilitate the amyloidogenesis of human disease‐associated amyloids remains largely in the experimental realm. Despite biochemical evidence and *in vivo* experimental manipulations, direct observations in human incidences of diseases, such as PD and AD, are mostly correlative. In PD, curli‐producing Enterobacteriaceae within the gut microbiome were associated with people having a more severe form of the disease [155]. More recently, an enrichment of this taxa and of encoded *csgA* was observed generally in the PD‐associated gut microbiome [156]. Similar observations appear in the AD‐associated gut microbiome, with increased abundance of amyloid producing Enterobacteriaceae [157, 158]. Given the experimental data, it is tempting to speculate that amyloids produced in the gut microbiome by these enriched taxa may contribute to aspects of disease pathology, directly or indirectly.

Friedland and Chapman [159] coined the term “mapranosis” to describe this process of microbiota associate proteinopathy and neuroinflammation. A single exposure to a bacterial amyloid may not be sufficient to trigger an amyloidogenic cascade leading to disease. However, the constant interactions that would occur during colonization within the microbiome may increase the probability of a cross‐seeding or misfolding event and initiate a pathogenic ratcheting of amyloid pathology. Age‐dependence observed in some neurodegenerative diseases may also play a role by allowing increased microbial‐derived molecules, including microbial amyloids, across epithelial and endothelial barriers in the GI tract and CNS. In fact, a recent study demonstrated a synergistic effect of a high‐fat diet (leading to increased intestinal permeability) and curli production on synuclein pathology [160].

If microbial amyloids are capable of initiating or contributing to amyloid pathologies linked to neurodegeneration, they would represent a potential tangible target for modification. Increased abundances of microbes that produce amyloids may be a risk factor for more rapid progression of some amyloid disease. Decreasing the abundance of amyloid producing bacteria in the GI tract through fecal microbiome transplant may represent one such pathway. Dietary interventions or probiotic‐type supplementation may select for certain species that outcompete certain amyloid‐producing microbes. Such interventions might be ideal for those at risk of amyloid disease to lengthen the time to disease conversion. Small molecules, including epigallocatechin gallate have demonstrated ability to inhibit the amyloid formation of microbial and mammalian amyloids [161, 162], as well as prevent the interaction between curli and ⍺Syn [115]. Microbiome‐targeted pharmaceuticals have emerged in the cardiovascular field, dampening enzymatic activity of certain microbial pathways that contribute to atherosclerosis and related disorders [163]. This line of enquiry may also prove fruitful to limit the production of microbial amyloids within the microbiome or their subsequent interactions with mammalian amyloids or proteostasis pathways.

Study of the precise connections between microbial amyloids (whether produced by rare infectious organisms or prevalent indigenous microbes) and human‐disease associated amyloids is poised to uncover key mechanisms to amyloid disease etiology and progression. While most of the data to date center around either the bacterial curli amyloid, the human ⍺Syn amyloid central to PD, or both, these may serve as foundations to uncover diverse and widespread interactions that occur at the interface of microbe and host and underlie or contribute to many amyloid diseases.

## Conflict of interest

The authors declare no conflict of interest.

## Author contributions

TS conceived and wrote this manuscript.
